# Early Post-stroke Activation of Vascular Endothelial Growth Factor Receptor 2 Hinders the Receptor 1-Dependent Neuroprotection Afforded by the Endogenous Ligand

**DOI:** 10.3389/fncel.2019.00270

**Published:** 2019-07-02

**Authors:** Alfredo Cárdenas-Rivera, Aura N. Campero-Romero, Yessica Heras-Romero, Andrés Penagos-Puig, Ruth Rincón-Heredia, Luis B. Tovar-y-Romo

**Affiliations:** ^1^Division of Neuroscience, Instituto de Fisiología Celular, Universidad Nacional Autónoma de México, Mexico City, Mexico; ^2^Microscopy Core Unit, Instituto de Fisiología Celular, Universidad Nacional Autónoma de México, Mexico City, Mexico

**Keywords:** Axitinib, ischemia, MCAO, stroke, SU1498, VEGF, VEGFR1, VEGFR2

## Abstract

Vascular endothelial growth factor (VEGF) has long been connected to the development of tissue lesion following ischemic stroke. Contradictory findings either situate VEGF as a promoter of large infarct volumes or as a potential attenuator of damage due to its well documented neuroprotective capability. The core of this discrepancy mostly lies on the substantial number of pleiotropic functions driven by VEGF. Mechanistically, these effects are activated through several VEGF receptors for which various closely related ligands exist. Here, we tested in an experimental model of stroke how the differential activation of VEGF receptors 1 and 2 would modify functional and histological outcomes in the acute phase post-ischemia. We also assessed whether VEGF-mediated responses would involve the modulation of inflammatory mechanisms and how this trophic factor acted specifically on neuronal receptors. We produced ischemic infarcts in adult rats by transiently occluding the middle cerebral artery and induced the pharmacological inhibition of VEGF receptors by i.c.v. administration of the specific VEGFR2 inhibitor SU1498 and the pan-VEGFR blocker Axitinib. We evaluated the neurological performance of animals at 24 h following stroke and the occurrence of brain infarctions analyzed at the gross metabolic and neuronal viability levels. We also assessed the induction of peripheral pro- and anti-inflammatory cytokines in the cerebrospinal fluid and blood and assessed the polarization of activated microglia. Finally, we studied the direct involvement of cortical neuronal receptors for VEGF with *in vitro* assays of excitotoxic damage. Preferential VEGFR1 activation by the endogenous ligand promotes neuronal protection and prevents the presentation of large volume infarcts that highly correlate with neurological performance, while the concomitant activation of VEGFR2 reduces this effect, even in the presence of exogenous ligand. This process partially involves the polarization of microglia to the state M2. At the cellular level, neurons also responded better to the preferential activation of VEGFR1 when challenged to *N*-methyl-D-aspartate-induced excitotoxicity. Endogenous activation of VEGFR2 hinders the neuroprotective mechanisms mediated by the activation of VEGFR1. The selective modulation of these concurrent processes might enable the development of therapeutic approaches that target specific VEGFR1-mediated signaling during the acute phase post-stroke.

## Introduction

The neuroprotective molecular mechanisms that drive endogenous adaptive responses to injury in the central nervous system (CNS) are mostly unknown, and their elucidation would ideally open new avenues for rehabilitation. A very promising process to target therapeutically is driven by vascular endothelial growth factor A (VEGF), which has long been implicated in the regulation of several events that take place following ischemic stroke, the leading cause of acquired disability in the developed world.

VEGF regulates a series of molecular processes that allow tissue to adapt to the conditions that prevail after stroke, such as neurovascular remodeling and repair, neuroprotection, brain plasticity and the recruitment and proliferation of neuronal precursors (Zhang et al., 2000; Ma et al., 2012; Dzietko et al., 2013; Greenberg and Jin, 2013). However, VEGF is also responsible for some damaging processes, such as cerebral edema and exacerbated blood-brain barrier leakage (Eliceiri et al., 1999; van Bruggen et al., 1999; Paul et al., 2001; Weis and Cheresh, 2005; Kim et al., 2018; Wu et al., 2018). Such opposing findings have, thus far, impeded the utilization of VEGF in a clinical setting aimed at alleviating the sequels of ischemic stroke.

VEGF acts by activating tyrosine kinase receptors 1 (VEGFR1, also known as FMS-like tyrosine kinase; Flt1) and 2 (VEGFR2, also known as kinase domain receptor; KRD, and fetal liver kinase 1; Flk-1). Both receptors are structurally closely related, each composed with seven immunoglobulin-like domains on the extracellular portion, a transmembrane motif, and an intracellular tyrosine kinase domain with conserved tyrosine phosphorylation sites (Grassot et al., 2006; Rahimi, 2006; Simons et al., 2016), although the ultimate biological actions driven by these receptors individually are somewhat different. Traditionally, VEGFR2 has been considered the canonical receptor for VEGF (Geiseler and Morland, 2018), and for a very long time, VEGFR1 has been thought to function as a decoy signal that counter-regulates VEGFR2 actions by sequestering the ligand, thus reducing its availability to binding VEGFR2 (Park et al., 1994; Meyer et al., 2006). Also, VEGFR1 is preferably activated by other members of the VEGF family, such as VEGF-B (Olsson et al., 2006).

An open question in all these molecular events is whether VEGF can also modulate the neuroinflammation elicited by ischemia (Geiseler and Morland, 2018). Neuroinflammation is an important component of the pathophysiology of stroke and other neurodegenerative processes where microglia, the resident macrophages of the CNS, play a central role.

Here we studied the mechanisms of VEGF-mediated neuroprotection in the acute phase of stroke using an *in vivo* model produced by the transitory occlusion of the middle cerebral artery in the rat (MCAO). The i.c.v. administration of VEGF in the early phase after stroke results in a significant reduction of infarct volume and increased neuronal survival. We found that if VEGFR1 gets preferably activated when VEGFR2 is inhibited, there is a reduction of infarct volume and edema, and an increase of neuronal survival and neurological outcome. Given the role of VEGFR1 on microglial responses to altered brain homeostasis, the underlying mechanisms of the VEGFR1-mediated protection partially involve also the modulation of the inflammatory response and microglial polarization to a neuroprotector phenotype. These results point toward VEGFR1 as an attractive therapeutic target for stroke.

## Materials and Methods

### Animals

In this study, we used young (1.5 months old; 270–290 g) Wistar rats that were subjected to MCAO as described below. Animals were housed in individual cages in a 12 h light/dark cycle with food and water *ad libitum*. All rats were killed at 24 h during the acute phase post-stroke. All experimental procedures were conducted under the current Mexican law for the use and care of laboratory animals (NOM-062-ZOO-1999) with the approval of the Institutional Animal Care and Use Committee (CICUAL-IFC-LTR93-16).

### Study Design

Animals were randomly divided into seven groups with an *n* = 10–13 for MCAO groups and 7 shams. The sample size was calculated *a priori* to detect a medium Cohen’s *d* effect size > 0.3, β power of 0.8 and significance of 0.05. Mortality rate was assumed to be 0.4 based on pilot experiments. These parameters were chosen to reduce the number of animals used. Inclusion criteria in analyzes considered the reduction of blood perfusion below 50% of basal values, which roughly corresponds to the effect of occluding the common carotid artery, no immediate recovery of reperfusion (above 50% baseline values within 3 min), total occlusion time between 90–95 min, absence of subarachnoid or intraparenchymal hemorrhages and survival at 24 h after stroke. This study is limited to assess effects on males.

### MCAO

Rats were put under isoflurane anesthesia (5% for induction followed by ≤1.5% during surgery) with oxygen as the carrier. Normal ventilation was autonomously maintained. Focal cerebral ischemia was induced with the Longa method (Longa et al., 1989) using a nylon monofilament with a silicone-dipped tip (403734, Doccol, Sharon, MA, United States) that was inserted in a stump created by cutting the ligated left external carotid artery and intraluminally advanced through the internal carotid artery until it reached and occluded the MCA at its inception in the Circle of Willis. MCA occlusion was kept for 90 min after which the monofilament was removed. Body temperature was maintained at 37°C with a heating pad for the duration of surgery. At the end of the procedure, the skin of the neck was sutured, and rats were returned to their cages. During the entire experimental procedure, the cerebral blood flow (CBF) was monitored in the territory irrigated by MCA with laser-Doppler flowmetry. For this, the parietal skull bone was thinned using a small mototool drill bit (Dremel, Racine, WI, United States) and a probe holder was glued onto it at stereotaxic coordinates (AP -1.5 L +3.5 from Bregma). A laser-Doppler probe (model 407, Perimed, Järfälla, Sweden) was inserted into the holder and connected to a Periflux System 5010 (Perimed). CBF was continuously monitored with an acquisition interval of 0.3 s using the Perisoft software (Perimed).

### Administration of VEGF and Pharmacological Inhibitors

VEGF and the VEGFRs inhibitors were administered by intracerebroventricular (i.c.v.) injections in the corresponding animal groups 30 min after intraluminal filament removal, which marked the beginning of reperfusion, with the following stereotaxic coordinates: AP -0.8 and L -1.5 from Bregma and V -4 from dura matter. Injections were performed with graduated glass microcapillary pipettes that were pulled to produce a tip < 50 μm in diameter (McCluskey et al., 2008) at a flux rate of 0.8 μL/min. Because of the non-polar nature of the inhibitors used in this study, we injected a corresponding volume of DMSO in all experimental groups to control for DMSO possible effects. Control sham- and MCAO-operated animals were injected with vehicle solutions consisting of 2 μL DMSO followed by 2 μL 0.1% BSA (4 μL final volume). Recombinant rat VEGF-A_164_ (Sigma) was prepared in a 0.1% BSA at a final concentration of 25 ng/μL, 2 μL VEGF were injected followed by 2 μL DMSO. SU1498 and Axitinib (both from Sigma) were prepared in DMSO at a concentration of 6.4 mM. Two μL of each inhibitor were administered in the corresponding groups followed by 2 μL 0.1% BSA or 25 ng/μL VEGF depending on the experimental condition.

### Infarct Volume Calculation

Animals were sedated with sodium pentobarbital and transcardially perfused with 200 mL 0.9% NaCl at 4°C, after which brains were collected and immediately sectioned into seven coronal slices (2 mm thick) that were incubated in 2% 2,3,5-triphenyltetrazolium chloride (TTC) at 37°C for 10 min. Images of sections were digitally acquired with a Lexmark scanner (Lexmark X2650, Lexmark, Canada) using Imaging Studio software (Lexmark, Canada). The infarcted and individual hemisphere areas of each section were measured with a semi-automatized macro routine executed in Fiji (Fiji-ImageJ, version 1.52i, NIH, United States) (Schindelin et al., 2012). Infarct volume was then calculated and corrected for edema with the following formula:

$$IV=\sum_{i=1}^{n}\left[\left(\frac{clHA_{i}\times IA_{i}}{ilHA_{i}}\right)t\right]$$

Where I*V* is the volume of infarct in mm^3^, *n* is the number of sections, *cl*H*A* is the area of the contralateral hemisphere in mm^2^, I*A* is the area of the damaged tissue in mm^2^, *il*H*A* is the area of ipsilateral hemisphere in mm^2^, and *t* is the thickness of each section in mm.

### Post-stroke Neuronal Viability Evaluation

For histological analyses, three rats per group were transcardially perfused with 200 mL ice-cold 0.9% NaCl followed by 250 mL ice-cold 4% paraformaldehyde (PFA). Brains were collected and post-fixed in 4% PFA for 24 h and then cryoprotected in 30% sucrose. Whole PFA-fixed brains were cut into 40 μm thick sections in a cryostat to produce 10 series of consecutive sections that were 400 μm apart. An entire series was mounted on gelatin-coated slides and stained with 1% cresyl violet. Digital images of a section series were acquired in an Olympus IX71 microscope with a 20X magnification using a 12 Megapixel Evolution UVF camera (Media Cybernetics, Buckinghamshire, United Kingdom). Images were processed in Image-Pro Plus 6.0 (Media Cybernetics). Soma diameters and shapes of pyramidal neurons within an area of 250 × 350 μm in layers IV and V of the somatosensory cortex in 3 consecutive sections were analyzed with an automatized macro routine executed in Fiji. Healthy cells were defined by circularity values of 0.47 – 0.88 and area of 30 – 250 μm^2^. Neuronal viability is reported as a survival index, which was calculated from each count of ipsilateral live neurons expressed as a proportion of the count in the respective contralateral area. The resulting values were normalized to the pooled indexes of the sham group. This algorithm is calculated with the following formula:

$$\mathrm{SI}=p\left({\sum_{1}^{t}\left(\frac{\sum_{i=1}^{n}\left(C_{i}^{il}/C_{i}^{cl}\right)}{n}\right)}_{t}\right)/t\left({\sum_{1}^{p}\left(\frac{\sum_{0=1}^{m}\left(S_{o}^{il}/S_{o}^{cl}\right)}{m}\right)}_{p}\right)$$

Where SI is the survival index, *C^il^* is the number of live neurons in the ipsilateral side, *C^cl^* is the number of live neurons in the respective confraternal side, *n* is the number of coronal sections analyzed for each brain, *t* is the number of animals analyzed per experimental group, *S^il^* is the number of neurons in the left side of the sham group, *S^cl^* is the number of neurons in the right side of the sham group and, *m* is the number of sections analyzed for each sham brain and *p* is the number of sham rats. No statistical differences were detected in absolute numbers of live neurons among contralateral sides across experimental groups.

### Behavioral Testing

Animals were evaluated with a battery of neurological tests to assess sensorimotor deficits 24 h after stroke. The severity of functional deficits was scored by assessing eight items described in Table 1 with slight modifications from previous reports (Mattsson et al., 1997; Schallert et al., 2000; Burguete et al., 2006; Zhang et al., 2015). Maximum score (32 total) was assigned to animals without neurological deficits. All evaluations were cross-validated by a trained observer blinded to the experimental treatment that analyzed recorded videos of the tests.

**Table 1:** Items evaluated in neurofunctional assessments.

Neurological assessment	Reflex	Score	Previously reported
Spontaneous activity	Exploring an open arena more than 20 s	3	Burguete et al., 2006
	Exploring between 10 and 20 s	2	
	Exploring less than 10 s	1	
	Not exploring or moving only when stimulated	0	
Contralesioned- wise circling	None	3	Burguete et al., 2006
	Spontaneous circling	2	
	Stimulus-induced circling	1	
	Not moving	0	
Prehensile grip of forepaws to a wire	Symetrical grip	4	Mattsson et al., 1997
	Asymetrical, preferably use of non-lesioned forelimb	3	
	Asymetrical, unable to hold body weight	2	
	Unable to hold grip with lesioned forelimb	1	
	Fall from wire	0	
Ability to rise while suspended from the tail	Unskewed side to rise	4	Zhang et al., 2015
	Rise preferably to the non-lesioned side	3	
	Able to rise only until reached the horizontal plane	2	
	Unable to rise	1	
	Unresponsive while suspended	0	
Cylinder test	Steading with both forelimbs at even height	4	Schallert et al., 2000
	Unable to bring lesioned forelimb to equal height of contralesioned forelimb	3	
	Does not support body weight on lesioned forelimb	2	
	Unable to rear	1	
	Unresponsive	0	
Protective retraction of forelimbs after poking	Symmetrical flex and move from the site	4	NA
	Asymmetrical flex of the ipsilateral forelimb and move from the site	3	
	Asymmetrical flex of the ipsilateral forelimb but stays on site	2	
	Slight movement of ipsilateral forelimb and stays on site	1	
	Does not flex forelimb and stays on site	0	
Body posture	Balanced	5	NA
	Head tilted to the right (lesioned flank)	4	
	Head tilted to the right and forearm extended	3	
	Body tilted to the right	2	
	Unable to keep posture with lesioned hindlimb	1	
	Unable to stand from lay down position	0	
March coordination	Symetrical movement	5	NA
	Forepaws extended during march	4	
	Support on ulnar side of lesioned forelimb	3	
	Dragging fingers during march	2	
	Dragging lesioned forelimb on dorsal side	1	
	Does not stand on lesioned forelimb	0	


*The neurological performance of every individual was assessed 24 h after stroke in eight items, and for each one, a corresponding value was assigned. Behavioral evaluations were cross-validated by a trained analyst blind to the experimental conditions.*

### Peripheral Cytokines Determination

Blood samples were obtained by cardiac puncture right before transcardial perfusion to determine circulating cytokine levels 24 h after stroke. Plasma was prepared from blood by adding 50 μL/mL 0.5 M EDTA and samples were stored at -80°C until used. Cytokine detection was carried out with a custom-made rat cytokine/chemokine magnetic bead Milliplex MAP panel (Millipore, Temecula, CA, United States) following the manufacturer instructions. A 96-well plate was used to simultaneously determine the concentrations of IL-1β, IL-4, IL-6, IL-10, IL-12p70, IL-17, TNF-α, CCL11, INFγ, CX3CL1, MIP-1α, and VEGF. The plate was read on a MAGPIX system (EMD Millipore, Darmstadt, Germany). Results were analyzed using the xPONENT software (Luminex, Madison, WI, United States). Standards of 200–16,000 pg/mL were used for generating the corresponding concentration curve of each analyte. Determinations were done in duplicates per biological replicate (*n* = 3).

### Cytokine mRNA Expression

Cytokine mRNA levels were determined 24 h after stroke by real-time quantitative PCR (RT-qPCR). For this, 1 mL of blood was combined with 50 μL 0.5 M EDTA and 10 mL of red blood cells lysis buffer (155 mM NH_4_Cl, 12 mM NaCO_3_, 0.1M EDTA, pH 7). Samples were centrifuged at 1,900 × *g* for 8 min at RT in a Sorvall ST8 centrifuge (Thermo Fisher Scientific. Karlsruhe, Germany) to obtain white blood cells pellet. Total RNA was isolated with 5:1 TRIzol (Ambion Life Technologies. Austin, TX, United States)-chloroform by phase separation centrifugation at 12,000 × *g* for 15 min at 4°C. RNA was precipitated in 500 μL isopropanol and washed in 75% ethanol. After air-drying, the pellet was reconstituted in RNAse and DNAse free ultra-pure water (Thermo Scientific). RNA concentration and purity were assessed using OD 260/280 ratios and gel electrophoresis. RT-qPCR was performed with One-step NZYSpeed RT-qPCR Green kit, ROX (nzyTECH, Lisbon, Portugal) according to the manufacturer directions. RT-qPCRs were run in 96-well plates in an Applied Biosystems StepOne RT-qPCR System (Thermo Fisher) with the following parameters: 20 min reverse transcription at 50°C, 2 min cDNA denaturing at 95°C, 40 cycles of 5 s denaturation at 95°C, 30 s annealing/amplification at 61°C. Relative quantification of gene expression was performed using the 2^-ΔΔC_T_^ method, with glyceraldehyde 3-phosphate dehydrogenase as a reference gene. Melting curve analyzes and gel electrophoresis evaluation of the RT-qPCR products were routinely performed to determine the specificity of the RT-qPCR reaction. Primer information for each gene is contained in Table 2.

**Table 2:** Primer sequences for RT-PCR analyses of cytokine transcripts.

Gene	Primer sequence	Amplicon length (bp)	Accession number
IL-1b	F: 5′-CCCTGCAGCTGGAGAGTGTGG-3′	153	NM_031512.2
	R: 5′-TGTGCTCTGCTTGAGAGGTGCT-3′		
IL-6	F: 5′-CGAGCCCACCAGGAACGAAAGTC-3′	84	M26744.1
	R: 5′-CTGGCTGGAAGTCTCTTGCGGAG-3′		
TGFb	F: 5′-ACCTGCAAGACCATCGACAT-3′	154	NM_021578.2
	R: 5′-TGTTGTACAAAGCGAGCACC-3′		
GAPDH	F: 5′-GCATCTTCTTGTGCAGTGCC-3′	278	NM_017008.4
	R: 5′-GATCTCGCTCCTGGAAGATGG-3′		


### Immunohistochemistry and Confocal Microscopy

PFA-fixed brain sections containing infarct core and penumbra were blocked with 5% bovine serum albumin in Tris-buffered saline with 0.5% v/v Triton X-100 (TBS-T) and incubated with anti-Iba-1 (1:200; Wako, Richmond, VA, United States) and anti-arginase-1 antibodies (1:200; Invitrogen) for 48 h. Sections were washed three times with TBS followed by 2 h incubation at RT with Alexa Fluor 488-conjugated anti-mouse and Alexa 546-conjugated anti-rabbit antibodies (1:2000 each; Invitrogen, Carlsbad, CA, United States) in TBS. Images were obtained in a Zeiss LSM 800 confocal microscope using a 63X objective. An average of 45 optical slices was obtained every 0.5 μm for each Z-stack.

### Cortical Neuronal Cultures

Cortical neuronal cultures were prepared from embryonic day 17 Wistar rats as previously described (Tovar-y-Romo et al., 2012). Briefly, cortices were isolated and trypsinized, and cells were dissociated by trituration in a Ca^2+^ and Mg^2+^ free Hanks’ balanced salt solution (Gibco, Carlsbad, CA, United States). Neurons were plated at a density of 1.3 × 10^5^ cells/cm^2^ in polyethyleneimine-coated 24-well plates in Neurobasal medium supplemented with B-27 (Gibco) and 1% antibiotic/antimycotic solution (104 U of penicillin G/ml, 10 mg of streptomycin/ml, and 25 μg of amphotericin B/ml) (Sigma). Ten μM cytosine β–D-arabinofuranoside (Sigma) was added on DIV 3 to prevent the proliferation of astrocytes.

Immunofluorescent staining was performed in cells plated on glass coverslips and fixed with 100% methanol for 10 min at -20°C on DIV 7. Neurons were incubated with anti-VEGFR1 or anti-VEGFR2 (1:100 each, LifeSpan Biosciences, Seattle, WA, United States) and anti-microtubule-associated protein 2 (MAP2; 1:100, Sigma) overnight at 4°C. Labeled cells were incubated with Alexa Fluor 546- and Alexa Fluor 488-conjugated antibodies (1:200 each, Life Technologies), for 1 h at RT, after three washes, neurons were incubated with DAPI for 5 min and mounted on glass slides. Cells were imaged with confocal microscopy as described above with a 40X objective. An average of 30 optical slices was obtained every 0.5 μm for each Z-stack.

### Cortical Neurons Excitotoxicity Assays

Neuronal cultures were challenged on DIV 11 with 1, 10, or 100 μM NMDA + 1 μM glycine for 24 h to induce excitotoxic damage or neuronal death. Rat recombinant VEGF-A_164_ was added to some experimental wells at a concentration of 10 ng/mL for 24 h. In some experiments, we added, 2 μM SU1498 or 2 μM Axitinib for the same time. Neuronal viability was inferred from metabolic activity determined by standard 3-(4,5-dimethylthiazol-2-yl)-2,5-diphenyltetrazolium bromide (MTT) reduction. For this, neurons were incubated with 0.1 mg/mL MTT for 2 h at 37°C at the end of the analyzed period. Media was removed, and formazan precipitations were dissolved in a 4 mM HCl isopropanol solution. The absorbance of cell debris-free supernatants was read in a spectrophotometer (Beckman Coulter) with a 570 nm wavelength. Data are presented as percentage relative to the absorbance of control conditions. In some experimental conditions we corroborated neuronal viability with the life/dead fluorescence assay (Thermo Fisher Scientific) that stain live cells with calcein AM (green) and dead cells with ethidium homodimer-1 (red), following manufacturers directions.

In an independent series of experiments, neurons were subjected to the same conditions and dendritic spines were stained with the Neurite Outgrowth Staining kit (Life Technologies, Carlsbad, CA, United States) following the manufacturer directions. Briefly, neurons plated on glass coverslips were fixed with 3.7% PFA for 30 min at RT, and 1X bright orange-fluorescent dye was added to stain the outer cell membrane surfaces. After incubation with a background suppression solution, coverslips were mounted on glass slides with a permanent mounting medium with antifading agents (DAKO, Santa Clara, CA, United States). Dendritic branches were visualized using a Leica DM1000 microscope under epifluorescence illumination (568-nm excitation and 580-nm emission) with a 40X objective. A minimum of 10 neurons from 3 independent experiments was analyzed for each condition. The number of dendritic spines, defined as thin protrusions emerging from dendritic processes, extending from two to five primary dendrites/neuron was quantified for a distance of approximately 20 μm from the cell soma. Neurite lengths and spine numbers were quantified with ImageJ (NIH, United States). Spine density across all measured dendritic segments was normalized to the length of the primary dendrite.

### Statistics

R- Feather Spray version 3.5.1, (R Foundation for Statistical Computing, Vienna, Austria) was used to analyze all data from *in vivo* experiments. Normal distribution in each data set was corroborated using the Shapiro–Wilk normality test. Data were tested by one-way analysis of variance (ANOVA) followed by Tukey *post hoc* test. Data were considered significant at α ≤ 0.05 level. All data are shown as mean ± 2SD. For *in vitro* experiments without normal distribution we did a Kruskal–Wallis, followed by Dunn *post hoc* test. Results are expressed as mean ± SEM.

## Results

### VEGF Mediates Neuroprotection in the Acute Phase of Stroke

**FIGURE 1:**
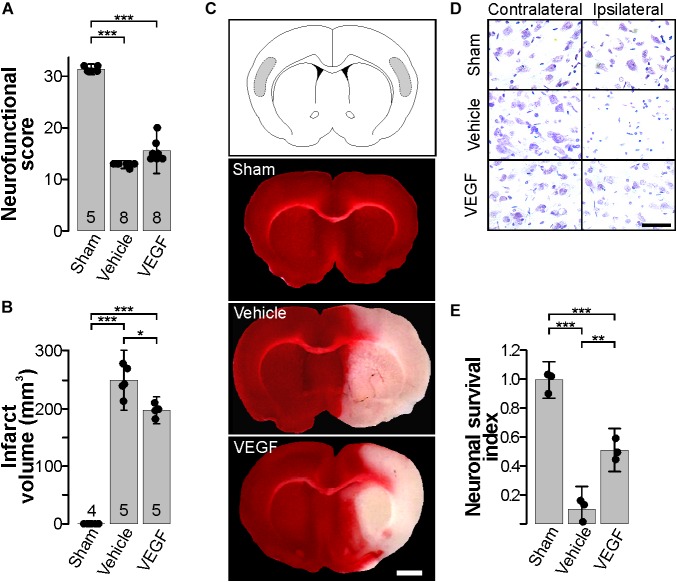
Exogenous VEGF administration in the acute phase post-stroke is neuroprotective. **(A)** Behavioral performance of sham-operated animals and stroked rats treated with vehicle or VEGF 24 h post-stroke. Animals were assessed individually in 8 items with scoring values that added up to 32 points that corresponded to neurologically unaffected performance, see Table 1. **(B)** Mean infarct volume ± 2 SD of the indicated experimental groups. In **(A,B)**, the number of experiments analyzed in each group is indicated at the bottom of each column; individual data points are plotted for each group. **(C)** Representative images of thick coronal sections of brains stained with TTC from a sham-operated animal and stroked rats treated with vehicle or VEGF. The unstained (pinkish) portion of the tissue depicts the infarct. Images show brain structures at around Bregma level in the anteroposterior axis. Bar equals 2 mm. **(D)** Representative photomicrographs of Nissl-stained thin sections of the cortical areas shaded in the diagram in (top **C**). Alive neurons have a pyramidal morphology and cytosolic light violet stain, while damaged neurons appear as condensed pycnotic nuclei with enhanced concentration of the dye. Bar equals 50 μm. **(E)** Survival index represents the portion of alive neurons present at 24 h post-stroke relative to the same region in the corresponding contralateral side normalized to the number of alive neurons in sham-operated animals. ^∗^*p* < 0.05, ^∗∗^*p* < 0.01, and ^∗∗∗^*p* < 0.001 in one-way ANOVA followed by Tukey *post hoc* test.

There are contradictory findings on the neuroprotective effects of VEGF in stroke. Most of the reported discrepancies can be grounded on time, dose and route of administration of exogenous VEGF in several models of stroke (Ma et al., 2012; Tovar-y-Romo et al., 2016). Because of this, we first tested the effect of administering a single bolus of 50 ng rat recombinant VEGF-A_164_ by i.c.v. injection 30 min after the beginning of reperfusion (Figure 1), the dose was chosen based on pilot experiments, and also on a previous study showing that this dose and method of administration is protective in other models of neurodegeneration (Tovar-y-Romo and Tapia, 2012). This protocol was carried out using a pulled glass microcapillary pipette to minimize mechanical damage in order to prevent the activation of inflammatory processes merely associated with the injection procedure. Twenty four hours after stroke, animals were evaluated in a series of neurobehavioral and motor tasks to determine the level of neurological alterations produced by the stroke (Figure 1A). Animals that were injected with vehicle only (DMSO + 0.1% BSA; 4 μl) showed a noticeable weakness and impaired movement of the lesioned side forelimb. These animals were unable to climb a grid and support their body weight and had a noticeable difficulty rising when placed on their sides, plus ∼60% of animals had epileptiform seizures, mostly represented by tonic-clonic convulsions, barrel rolls and running fits. Stroked rats also displayed signs of neuropathic pain when stimulated by slightly poking the affected flank. Administration of 50 ng recombinant VEGF at the beginning of reperfusion did not improve significantly the neurological deficits presented after stroke and also failed to prevent seizures.

Nonetheless, VEGF did reduce infarct volumes by about 20% in comparison to the group that received vehicle only (Figure 1B,C). Moreover, neuronal viability was also preserved as revealed by analyzing the somatosensory region of the parietal cortex, in which ∼50% neurons remain alive at 24 h post-stroke (Figure 1D,E). Therefore, with this experimental settings, we found a condition in which exogenous VEGF did produce a protective impact on brain tissue without translating into a better neurobehavioral outcome at this short time point after stroke.

### VEGFR2 Activation Hinders VEGFR1-Driven Neuroprotection Elicited by Endogenous Ligand in the Acute Phase Post-stroke

**FIGURE 2:**
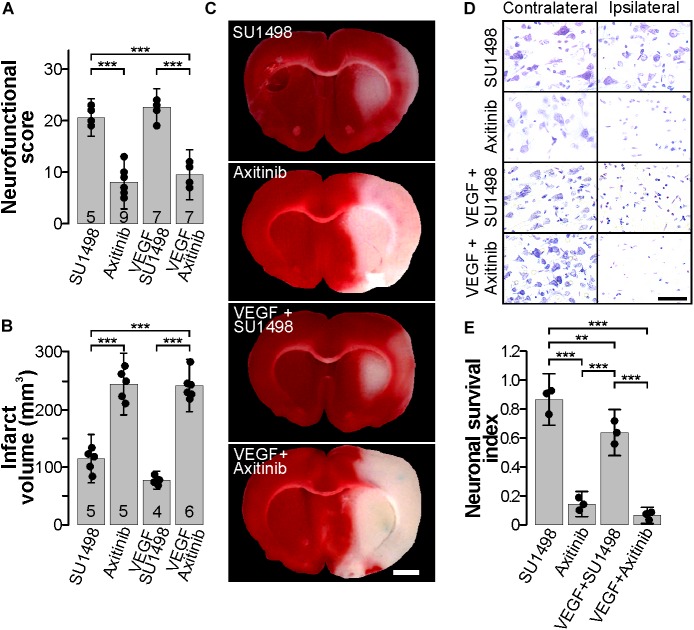
Blocking the VEGFR2 in the acute phase post-stroke is neuroprotective. **(A)** Behavioral performance of stroked rats treated with the VEGFR2 specific inhibitor SU1498 and the pan-VEGFR inhibitor Axitinib with and without exogenous VEGF, 24 h post-stroke. **(B)** Mean infarct volume ± 2 SD of the indicated experimental groups treated with SU1498 or Axitinib alone or in combination with exogenous VEGF. In **(A,B)**, the number of experiments analyzed in each group is indicated at the bottom of each column; individual data points are plotted for each group. **(C)** Representative images of thick coronal sections of brains stained with TTC from stroked animals treated with SU1498 and Axitinib with or without exogenous VEGF. The unstained (pinkish) portion of the tissue depicts the infarct. Images show brain structures at around Bregma level in the anteroposterior axis. Bar equals 2 mm. **(D)** Representative photomicrographs of Nissl-stained thin sections of cortical areas in the somatosensory region. Alive neurons have a pyramidal morphology and cytosolic light violet stain, while damaged neurons appear as condensed pycnotic nuclei with enhanced concentration of the dye. Bar equals 50 μm. **(E)** Survival index represents the portion of live neurons present at 24 h post-stroke relative to the same region in the corresponding contralateral side normalized to the number of live neurons in sham-operated animals. ^∗∗^*p* < 0.01 and ^∗∗∗^*p* < 0.001 in one-way ANOVA followed by Tukey *post hoc* test.

**FIGURE 3:**
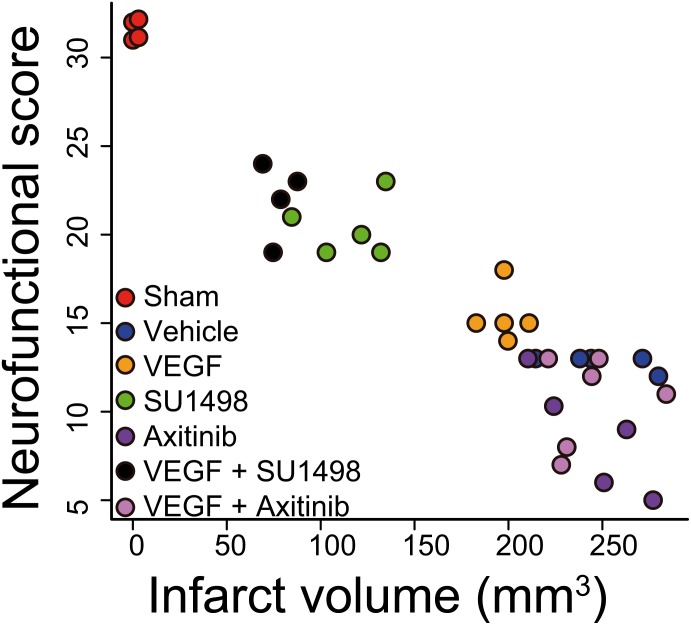
Activation of VEGFR2 hinders VEGFR1-dependent neuroprotection afforded by the endogenous ligand. The plot shows the correlation of neurofunctional score to the infarct volume produced by MCAO in animals injected i.c.v. 30 min after the beginning of reperfusion with vehicle, or exogenous VEGF alone or in combination with VEGFR2 specific inhibitor SU1498 or VEGFR general inhibitor Axitinib. Two groups of animals that received the inhibitors individually are also presented. Each data point represents the values obtained in a single animal. Groups segregate by experimental condition and produce a strong correlation with an adjusted *R*^2^ = 0.8747. Animals in which VEGFR2 early activation was prevented show the best levels of protection, both neurofunctional and histological, inhibiting VEGFR1 in addition to VEGFR2 blocked this effect.

It has been known for a long time that VEGF synthesis is upregulated by ischemia immediately after an experimental stroke (Hayashi et al., 1997; Plate et al., 1999; Hai et al., 2003), and this phenomenon has also been reported to occur in human patients (Issa et al., 1999). It is suspected that such VEGF increase is mechanistically involved in neuroprotection. Considering the large body of evidence that involves VEGFR2 participation in neuroprotection (Olsson et al., 2006; Tovar-y-Romo et al., 2016; Geiseler and Morland, 2018), we set out to determine whether VEGFR2 would be primordially responsible for the protection seen here. For this, we administered i.c.v. 13 nmoles of SU1498, a selective VEGFR2 inhibitor (Strawn et al., 1996), 30 min after the beginning of reperfusion and analyzed the overall effects of this procedure 24 h after stroke. Animals in this group displayed signs of neurological deficits mainly characterized by paresis of the lesioned forelimb. However, all animals in this group performed better in the neurological tests and showed significantly fewer alterations than rats that were administered with vehicle alone (Figure 2A, 3). Furthermore, co-administration of VEGFR2 antagonist together with a bolus of 50 ng exogenous agonist also resulted in a noticeable improvement in neurological function as compared to animals that received vehicle alone but also to the group that was administered with VEGF, which displayed better recoveries (Figure 2A, 3) – thus pointing to a VEGFR2-independent effect driven by the endogenous ligand.

Given the lack of a specific pharmacological inhibitor of VEGFR1, we employed the general VEGFR inhibitor Axitinib that blocks all VEGF receptors (Inai et al., 2004). The MCAO group that received 13 nmoles of Axitinib at 30 min of the beginning of reperfusion presented exacerbated neurological symptoms described above that were followed by a lethargic state in which animals laid on their right side. As expected, infarct volumes in this group were large and comparable to the vehicle group (Figure 1B,C, 2B,C). Finally, blocking all VEGFRs prevented any protection mediated by the i.c.v. administration of exogenous VEGF and animals displayed neurological impairments characteristic of large infarctions (Figure 2A–C). Brain damage seen in TTC stainings in this group was of proportions similar to the ones seen in the control untreated group (Figure 2B,C). Accordingly, neuronal death was reduced in the animals that received SU1498 alone or in combination with VEGF and was increased in animals treated with Axitinib (Figure 2D,E).

Correlating the neurological performance of animals 24 h after stroke to the extent of brain tissue damage allowed us to clearly segregate groups by experimental treatment and show that blocking VEGFR2 and potentiating the activation of VEGFR1 by the administration of exogenous ligand, afforded the best level of neuroprotection, closely followed by only blocking VEGFR2 (Figure 3). This analysis shows a strong correlation of *R*^2^ = 0.8747 with a *p* = 3.33 × 10^-16^. The sole administration of the exogenous ligand, while still protective, does not result in a very different outcome as compared to all the non-protected experimental conditions, in which we either not treated animals or inhibited all VEGFRs (Figure 3).

### VEGFR1-Mediated Neuroprotection Partially Involves M2 Polarization of Microglia With a Minimal Discernible Impact on Systemic Markers of Inflammation

**FIGURE 4:**
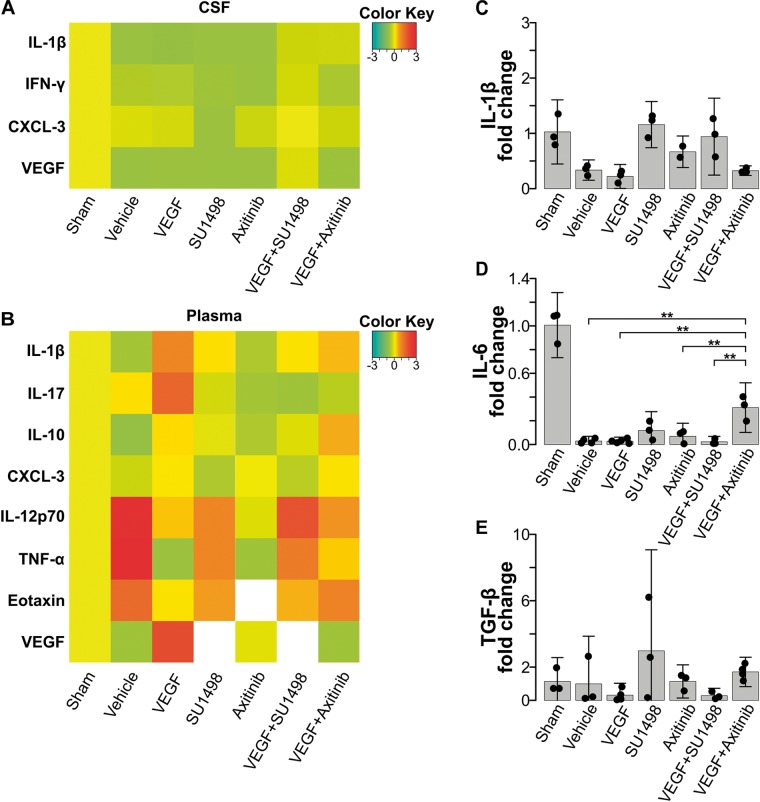
VEGFR1-dependent neuroprotection does not reflect on changes in peripheral inflammatory markers. **(A)** Heatmap of cytokines and VEGF detected in CSF 24 h after stroke. **(B)** Heatmap of peripheral cytokines and VEGF detected in plasma 24 h after stroke. No significant differences were detected in these markers in the acute phase following stroke. White colored areas are from groups with undetectable readings. Changes in transcriptional expression of **(C)** IL-1β, **(D)** IL-6, and **(E)** TGF-β, determined by RT-qPCR relative to GAPDH in blood 24 h following stroke. No differences were found among experimental groups.

VEGF drives the migration of macrophages and microglia to the damaged CNS through VEGFR1-mediated signaling (Forstreuter et al., 2002; Huang et al., 2013; Lelli et al., 2013). Therefore, we decided to study whether the neuroprotection exerted by the preferential activation of VEGFR1 would also involve the modulation of neuroinflammatory processes. We first assessed changes in inflammatory markers, both in CSF and in plasma, at 24 h post-stroke. We analyzed the content of IL-1β, IL-4, IL-6, IL-10, IL-12p70, IL-17, TNF-α, CCL11, INFγ, CX3CL1, and MIP-1α with a multiplex array for these cytokines in samples collected prior transcardial perfusion. None of the detected cytokines in CSF (IL-1β, INFγ, CXCL-3) showed a significant change compared to the values seen in the vehicle-treated group (Figure 4A). Likewise, for most of the peripheral markers of inflammation that produced a readable measurement in plasma, we did not find differences among experimental groups. However, we detected with this array that the inhibition of all VEGFRs with Axitinib blunted the release of peripheral markers of inflammation IL-12, TNFα, and CCL11. This blockade did not happen to the same degree when we selectively inhibited VEGFR2 (Figure 4B). Since under our experimental conditions we were not able to have reliable measurements of peripheral IL-1β, IL-6, and TGFβ, which have been previously identified as central players of the neuroinflammatory processes that happen after stroke, we decided to evaluate the levels of transcriptional expression of these cytokines in white blood cells collected 24 h after stroke. With RT-qPCR analyses, we determined that there are no significant changes in the level of transcription for neither of these molecules at the time point of our investigations and that VEGF or its receptors are not modifiers of these molecular responses (Figure 4C–E).

**FIGURE 5:**
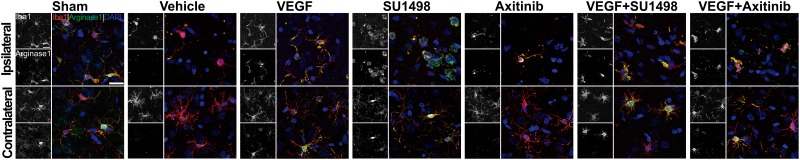
Blockade of VEGFR1 impedes the polarization of microglia to the M2 phenotype. Representative images of brain sections co-labeled with the microglia marker Iba-1 (red) and the M2 phenotype indicator arginase-1 (green). Images were taken from the somatosensory cortex adjacent to infarct core. Ameboid-like activated morphology is present under all MCAO conditions in the ipsilateral side. M2 polarizations are evident in most of the Iba-1 positive cells in the MCAO groups treated with vehicle, VEGF, SU1498 and the combination of VEGF and SU1498. Animals injected with the pan-VEGFR blocker Axitinib do not show labeling of M2 polarization in the activated microglia on the ipsilateral side. Bar equals 20 μm.

Next, we looked into the polarization of microglial cells to the anti-inflammatory phenotype M2, which is known to occur in the first 24 h after stroke (Hu et al., 2012). For this, we co-stained brain sections for the microglial marker Iba-1 and the M2 marker arginase-1. Strikingly, resting state morphologically looking microglia were found to express arginase-1 in sham animals at 24 h of transiently occluding the common carotid artery (Figure 5). In the control MCAO group, microglia in the lesioned cortical region looked morphologically activated, mainly with shortened cellular processes and rounded somas but cells in these regions lacked the expression of arginase-1. VEGF-treated animals, however, showed activated microglia expressing the M2 polarization marker and this was also the scenario in the groups treated with the VEGFR2 inhibitor SU1498 with and without VEGF. In the case of animals injected with Axitinib, we found fewer activated microglia expressing arginase-1, suggesting that VEGFR1 blockade also reduced the polarization of microglia to the anti-inflammatory phenotype.

### VEGF Acts Directly on Neurons Through the Activation of VEGFR1

**FIGURE 6:**
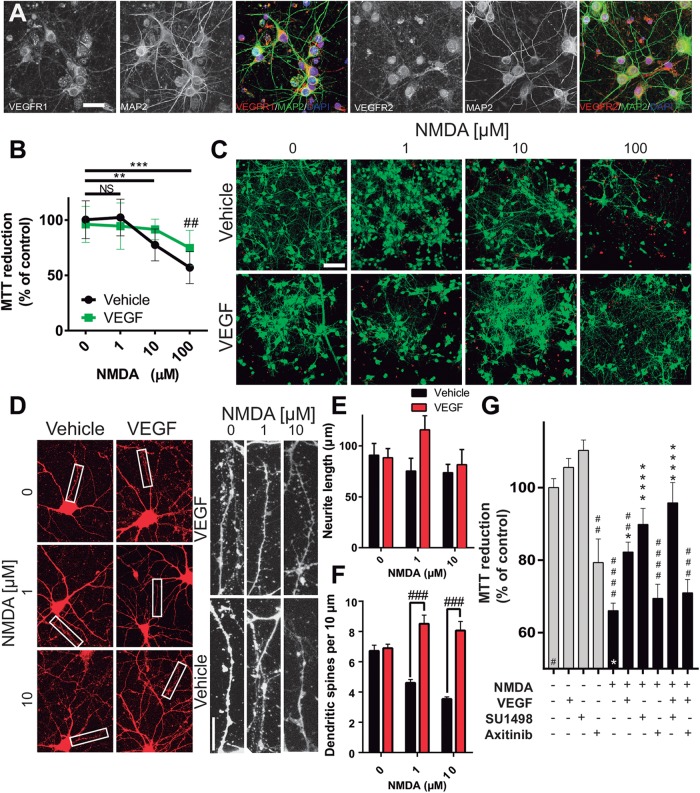
Neuronal VEGFR1 drives neuroprotection under excitotoxic stress. **(A)** Representative photomicrographs of cultured cortical neurons labeled with the neuronal marker MAP2 (green), and VEGFR1 and VEGFR2 (red). Bar equals 25 μm. **(B)** Neuronal survival assessed by MTT reduction of cultures exposed to increasing concentrations of NMDA for 24 h and 10 ng/mL VEGF. The graph shows the mean ± SEM of three independent experiments. **(C)** Representative images of cortical neurons exposed to increasing concentrations of NMDA for 24 h with or without 10 ng/mL VEGF, calcein stain labels alive neurons green, while dead cells are marked by the nuclear staining of bromide homodimer in red. Bar equals 200 μm. **(D)** Representative micrographs of neurons exposed to sublethal concentrations of NMDA for 24 h and stained with a membrane dye. VEGF preserved the structural integrity of neurites and dendritic spines. Images on the right are magnifications of the indicated areas on the left. Scale bar equals 10 μm. Quantifications of neurite length **(E)** and dendritic spines **(F)** of the experiments described in **(D)**. Each column represents the mean ± SEM of the neurites of 5 neurons analyzed in three independent experiments. **(G)** Neuronal survival assessed by MTT reduction of cultures exposed to 100 μM NMDA for 24 h and 10 ng/mL VEGF alone or in combination with 2 μM SU1498 and 0.2 μM Axitinib. The graph shows the mean ± SEM of three independent experiments. ^∗^*p* < 0.05, ^∗∗^*p* < 0.01, ^∗∗∗^*p* < 0.001, and ^∗∗∗∗^*p* < 0.0001 compared to NMDA alone, and ^##^*p* < 0.01, ^###^*p* < 0.001, and ^####^*p* < 0.0001 compared to untreated neurons in Kruskal–Wallis followed by Dunn *post hoc* test.

In the last part of our study, we analyzed whether the effects of preferentially activating VEGFR1 and the neuroprotection seen in the *in vivo* model of ischemic stroke were derived from the direct stimulation of neuronal receptors. Using an *in vitro* model of cultured cortical neurons we first determined the expression of both, VEGFR1 and VEGFR2 in these cells. Figure 6A shows the expression of both TRKs at 7 DIV, specifically in neurons. Expression of both receptors is clear in neuronal somas as well as in neurites. Then, we tested whether the addition of VEGF to neuronal cultures could rescue neurons from excitotoxicity, one of the primary mechanisms underlying neuronal death after ischemia. For this, we incubated the neurons with different concentrations of the glutamatergic agonist NMDA for 24 h in the same culture media in which cells were grown for 11 days. At the lowest concentration assayed (1 μM), neuronal death is not apparent and increasing the concentration of NMDA to 10, and 100 μM gradually increased the level of neuronal death (Figure 6B,C). Addition of 10 ng/mL recombinant VEGF reduced in about 20% the loss of metabolic activity in the neuronal cultures, which relates to the overall health of the cells and their survivability.

Even though the lower concentrations of NMDA tested here did not produce a substantial decrease of neuronal viability, neurons exposed to these conditions developed morphological signs of structural damage, mainly characterized by dendritic spine loss. Addition of VEGF to the culture media also prevented this morphological change and preserved spine structures although it did not modify the length of primary neurites (Figure 6D–F). With these experiments, we showed that VEGF executes trophic effects on cortical neurons to allow them to grow healthy in culture and make them less susceptible to excitotoxic death.

Finally, in an independent set of experiments, we tested the role of VEGFR1 and VEGFR2 in driving the pro-survival effects. Strikingly, blocking VEGFR2 activation by addition of SU1498 also resulted in a significant increase in neuronal protection, which was further potentiated by the co-administration of VEGF (Figure 6G). By contrast, inhibiting the activation of both receptors with Axitinib prevented entirely the neuroprotection exerted by VEGF (Figure 6G). These results replicated well the effects that we described first with the *in vivo* model, therefore, proving that the mechanisms of protection seen partially encompassed a direct involvement of neuronal receptors.

## Discussion

Since VEGF is a molecule with pleiotropic actions that can execute its functions by binding a number of different receptors, the exact role of VEGF increased expression after stroke in neuroprotective mechanisms has been the core of a controversy. The prevailing idea on the dual effects exerted by VEGF states that the dominant actions of this trophic factor, whether neuroprotective or detrimental, are chiefly dictated by the timing, dosage, and route of administration in experimental models of stroke (Wittko-Schneider et al., 2013; Geiseler and Morland, 2018). Moreover, it has been amply described that excessive levels of VEGF in the early phase following stroke promote BBB alterations and a general state that favors neurodegeneration. Interestingly, delayed administration of VEGF is more likely to result in neuroprotection (Sun et al., 2003; Dzietko et al., 2013), even though, others have also reported the protective effect of an early VEGF i.c.v. administration (Kaya et al., 2005).

In the brain, VEGFR1 and VEGFR2 are the most expressed receptors, with VEGFR1 showing a higher density in neurons (Yang et al., 2003; Du et al., 2010). Several reports have been published over the years indicating that VEGFR2 is the receptor predominantly responsible for neuroprotective actions, mainly by driving pro-survival signaling mediated by PI-3K/PKB and MEK/Erk pathways (Greenberg and Jin, 2013). Here, we found that the preferential activation of VEGFR1 by the endogenous ligand promotes neuronal protection and prevents the presentation of large volume infarcts; this is highly correlated with neurological performance. Of note, the concurrent activation of VEGFR2 prevents this effect, even in the presence of excess ligand. Strikingly, such protection still happens at the cellular level, where neurons are better protected when challenged to NMDA-induced excitotoxicity by the activation of VEGFR1 if VEGFR2 is blocked.

VEGF along with its receptors 1 and 2 are transcriptionally upregulated following stroke (Hayashi et al., 1997; Issa et al., 1999; Plate et al., 1999; Marti et al., 2000; Hai et al., 2003; Stowe et al., 2007, 2008) in a long-lasting fashion (Zhang et al., 2002), through the stabilization and subsequent binding of hypoxia-inducible factor 1 (HIF-1) to the hypoxia response element sequence in their promoters (Forsythe et al., 1996). Also, it has been shown that exogenous administration of VEGF causes the upregulation of VEGFR1 and VEGFR2 (Krum et al., 2008). Cellular distribution of VEGFRs might be key in understanding the temporal regulation of vasculature adaptations in the acute phase post-stroke. In this regard, the apicobasal localization of VEGFR1 and VEGFR2 is different, where VEGFR2 is predominantly expressed in the basal side and VEGFR1 in the apical pole that produces a differential response when the ligand acts at the circulation or the parenchymal sides of endothelial cells (Hudson et al., 2014). It has also been reported that the expression of VEGFR1 is upregulated while VEGFR2 is downregulated in endothelial cells following hypoxia, which changes the VEGFR1:VEGFR2 ratio altering the ultimate responses on cell viability and recovery (Ulyatt et al., 2011). It is also important to point out that the affinity of VEGFR1 for VEGF is about 1 order of magnitude higher than the one for VEGFR2 (K_d_ 2–10 pM for VEGFR1 and 75–250 pM for VGFR2) (de Vries et al., 1992; Ferrara, 2004; Shibuya, 2006). Although VEGFR3 is expressed in the adult rat brain (Hou et al., 2011), we ruled out the participation of this receptor in the neuroprotection seen here, even when it can also be inhibited by Axitinib, because it does not get activated by VEGF-A (Olsson et al., 2006; Leppänen et al., 2013) and its expression is primarily restricted to lymphatic endothelial cells (Iljin et al., 2001), which have a very low relative abundance in the brain (Louveau et al., 2015).

Our observations lead to reaffirm the critical role of VEGFR1 in the endogenous neuroprotective mechanisms elicited after ischemic insults to the brain. In this regard, it is known that the deletion of VEGF-B, a specific ligand for VEGFR1, produces 50% larger infarcts in mice when subjected to the permanent occlusion of the MCA in comparison to WT animals, by inhibition of apoptotic executioner molecules (Li et al., 2008). Protein analysis identified a significant increase of VEGF-B in CSF and the ischemic hemispheres, with increased VEGFR1 activation that also correlated with an increase in Akt phosphorylation, whereas an increase in VEGF in the contralateral hemisphere correlates with a significant increase in vascular density 7 days post-stroke (Guan et al., 2011). VEGFR1 activation in pericytes promotes the formation of stable new vessels in a mechanistic way independent of endothelial cells, and also slightly attenuated inflammation when administered in the sub-acute phase post-stroke (Jean LeBlanc et al., 2018). In this sense, it has also been shown that PlGF, a specific ligand for VEGFR1, is the most efficient promoter of angiogenesis, which produces mature non-leaky new vessels (Luttun et al., 2002; Gaál et al., 2013).

The direct VEGFR1 activation in neurons might be a mechanism more relevant than promoting healthy angiogenesis (Poesen et al., 2008; Dhondt et al., 2011; Ishrat et al., 2018). In this regard, it is noteworthy that VEGF associates to neurons in infarct and peri-infarct brain regions in a non-human primate model of stroke (Stowe et al., 2007), and interestingly, such association can even be more relevant than the interaction of this growth factor with astrocytes (Shen et al., 2018). Our results, however, indicate that all these neuroprotective mechanisms driven by VEGFR1 are suppressed in the first hours following stroke by the activation of VEGFR2. The exact mechanisms underlying this phenomenon are not fully elucidated, but they might involve the suppression of endoplasmic reticulum stress and apoptosis (Feng et al., 2019).

VEGF has some interesting roles in regulating neuroinflammation. It has been shown that macrophages express more VEGF in response to disturbances in brain metabolic homeostasis, especially responding to decreased glucose incorporation (Jais et al., 2016), and microglia in chronically affected regions of CNS upregulate their expression of VEGF (Nikodemova et al., 2014). However, not much is known about the involvement of VEGF, or its receptors, in the polarization of macrophages/microglia under inflammation. Interestingly, very recent evidence indicates that the antiangiogenic VEGF isoform VEGF_164_b is capable of blocking VEGFR1 prompting the M1 polarization of peripheral macrophages (Ganta et al., 2019), although it is known that this form of VEGF has neuroprotective effects in the CNS (Beazley-Long et al., 2013). Also, M2-polarized peripheral macrophages in culture are known to upregulate their VEGFR1 mRNA expression (Melton et al., 2015), which in microglial cells could lead to the inhibition of the expression of the scavenger receptor A following stroke (Xu et al., 2017). With the determinations carried out in the present study, we were unable to obtain reliable readings of the levels of soluble mediators of inflammation poured on CSF and plasma, however, many of these markers, like IL-6, have been disregarded as important predictors of the outcome (Clark Wayne et al., 2000; Worthmann et al., 2010). Nonetheless, we could characterize the overall response of microglia polarizing to the anti-inflammatory phenotype under the experimental conditions that allow the activation of VEGFR1. More detailed analyses are required to characterize these responses at the molecular and cellular levels.

## Conclusion

We found that the activation of VEGFR2 in the first hours post-stroke obstructs endogenously-coded mechanisms of adaptation to injury in the brain. We propose here the existence of a VEGFR2-mediated antagonism of VEGFR1, which has not been reported previously. This process would constitute a fundamental mechanism of endogenous operations that drive adaptive responses in the brain after stroke that is worthy of further exploration. Our results also strengthen the notion that VEGFR1 has a critical role in neuroprotection, and that targeting this receptor would be useful at developing restorative therapies for stroke.

## Data Availability

The datasets used and/or analyzed during the current study are available from the corresponding author on reasonable request.

## Ethics Statement

This study was carried out in accordance with the Mexican law for the use and care of laboratory animals (NOM-062-ZOO-1999) with the approval of the Institutional Animal Care and Use Committee (CICUAL-IFC-LTR93-16).

## Author Contributions

AC-R and LT-y-R conceived the project and designed the experiments. AC-R conducted the *in vivo* experiments, wrote the R-based scripts to process data, and carried out statistical analyses. YH-R cross-validated the neurological assessments. ANC-R and AP-P conducted the *in vitro* experiments. RR-H was responsible for confocal microscopy acquisition and analyses. AC-R, ANC-R, YH-R, and LT-y-R analyzed the data. LT-y-R wrote the manuscript. All authors read and approved the final version of the manuscript.

## Conflict of Interest Statement

The authors declare that the research was conducted in the absence of any commercial or financial relationships that could be construed as a potential conflict of interest.
